# Vestibular pathways involved in cognition

**DOI:** 10.3389/fnint.2014.00059

**Published:** 2014-07-23

**Authors:** Martin Hitier, Stephane Besnard, Paul F. Smith

**Affiliations:** ^1^Inserm, U 1075 COMETECaen, France; ^2^Department of Pharmacology and Toxicology, Brain Health Research Center, University of OtagoDunedin, New Zealand; ^3^Department of Anatomy, UNICAENCaen, France; ^4^Department of Otolaryngology Head and Neck Surgery, CHU de CaenCaen, France

**Keywords:** vestibular system, neuroanatomy, cognition, hippocampus, vestibular cortex, spatial orientation, basal ganglia, theta

## Abstract

Recent discoveries have emphasized the role of the vestibular system in cognitive processes such as memory, spatial navigation and bodily self-consciousness. A precise understanding of the vestibular pathways involved is essential to understand the consequences of vestibular diseases for cognition, as well as develop therapeutic strategies to facilitate recovery. The knowledge of the “vestibular cortical projection areas”, defined as the cortical areas activated by vestibular stimulation, has dramatically increased over the last several years from both anatomical and functional points of view. Four major pathways have been hypothesized to transmit vestibular information to the vestibular cortex: (1) the vestibulo-thalamo-cortical pathway, which probably transmits spatial information about the environment via the parietal, entorhinal and perirhinal cortices to the hippocampus and is associated with spatial representation and self-versus object motion distinctions; (2) the pathway from the dorsal tegmental nucleus via the lateral mammillary nucleus, the anterodorsal nucleus of the thalamus to the entorhinal cortex, which transmits information for estimations of head direction; (3) the pathway via the nucleus reticularis pontis oralis, the supramammillary nucleus and the medial septum to the hippocampus, which transmits information supporting hippocampal theta rhythm and memory; and (4) a possible pathway via the cerebellum, and the ventral lateral nucleus of the thalamus (perhaps to the parietal cortex), which transmits information for spatial learning. Finally a new pathway is hypothesized via the basal ganglia, potentially involved in spatial learning and spatial memory. From these pathways, progressively emerges the anatomical network of vestibular cognition.

## Introduction

The vestibular system senses angular and linear acceleration of the head in three dimensions and is responsible for generating vestibulo-ocular and vestibulo-spinal reflexes that stabilize the visual image on the retina and adjust posture (respectively), during head movement. However, this sensory system also has a role in cognition. Anyone who has experienced vestibular-induced vertigo will admit that spatial perception and cognition dramatically change when the environment seems to be spinning around.

Research in both animals and humans has revealed the role of the vestibular system in cognition (see for review Smith et al., 2010): it concerns self-motion perception, bodily self-consciousness, spatial navigation, spatial learning, spatial memory and object recognition memory (Liu et al., 2004; Zheng et al., 2004; Angelaki and Cullen, 2008; Zheng et al., 2006, 2009; Baek et al., 2010; Besnard et al., 2012; Smith, 2012).

The anatomical substrates of these cognitive functions have been studied with neuronal tracers, electrophysiology, immunohistochemistry, functional imaging and also indirectly through behavioral studies. Four different pathways have been proposed to transmit vestibular information to cortical centers involved in cognition: (1) the vestibulo-thalamo-cortical pathway; (2) a pathway from the dorsal tegmental nucleus via the lateral mammillary nucleus, the anterodorsal nucleus of the thalamus to the entorhinal cortex; (3) a pathway via the nucleus reticularis pontis oralis, the supramammillary nucleus and the medial septum to the hippocampus; and (4) a possible pathway via the cerebellum, and the ventral lateral nucleus of the thalamus (perhaps to the parietal cortex) (see Smith, 1997; Smith et al., 2005; Hüfner et al., 2007 for reviews).

Nevertheless, some segments of these pathways are only hypothesized, with no real proof of vestibular input involved, while the validity of other segments is reinforced by recent studies (e.g., Aravamuthan and Angelaki, 2012; Clark et al., 2012; Shibata and Honda, 2012; Chen et al., 2013; Yakusheva et al., 2013).

**Figure 1:**
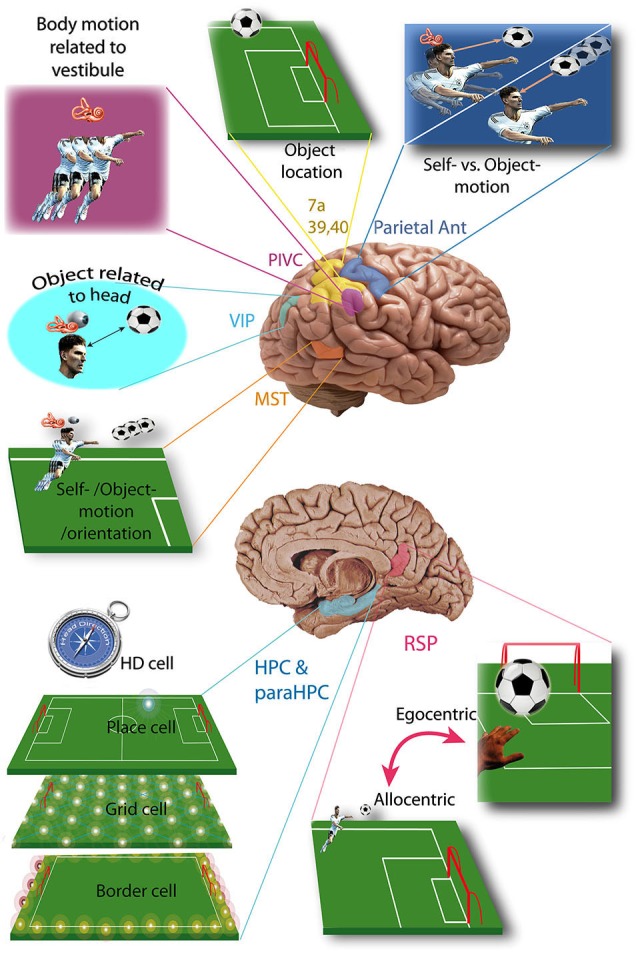
**Vestibular cortices and spatial cognition**. Vestibular cortices involved in spatial cognition, illustrated in a soccer player: HD cell, head direction cell; HPC, hippocampus; MST, medial superior temporal area; ParaHPC, parahippocampal cortex; Parietal Ant, parietal anterior cortex; PIVC, parieto-insular vestibular cortex; RSP, retrosplenial cortex; VIP, ventral intraparietal area; 7a, 39, 40 Brodmann area. (References in the text).

**Figure 2:**
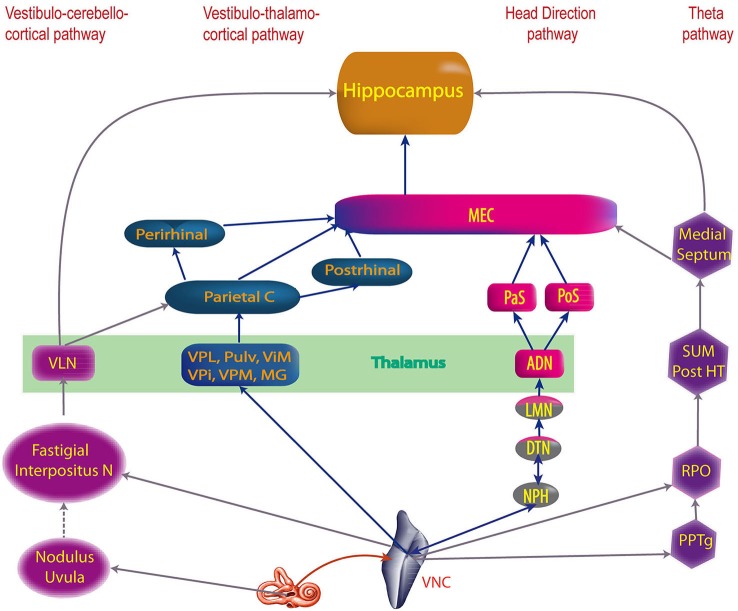
**The four main vestibular pathways to hippocampus**. ADN, anterodorsal nucleus of the thalamus; DTN, dorsal tegmental nucleus; Interpositus N, anterior and posterior interposed nuclei; LMN, lateral mammillary nuclei; MEC, medial entorhinal cortex; MG, medial geniculate nucleus; NPH, nucleus prepositus hypoglossi; Parietal C, Parietal cortex; PaS, parasubiculum; Perirhinal, Perirhinal cortex; PoS, posterior subiculum (i.e dorsal part of the presubiculum); Post HT, posterior hypothalamus; Postrhinal, postrhinal cortex; PPTg, pedunculopontine tegmental nucleus; Pulv, pulvinar; RPO, reticularis pontis oralis; SUM, supramammillary nucleus; ViM, ventralis intermedius nuclei of the thalamus; VLN, ventral lateral nucleus of the thalamus; VNC, vestibular nucleus complex; VPi, ventral posterior inferior nucleus of the thalamus; VPL, ventral posterior lateral nucleus of the thalamus; VPM, ventral posterior medial nuclei of the thalamus (built from multiple references: cf text).

Here we will review and analyze the current knowledge relating to the vestibular pathways involved in cognition. We begin by describing the cortical areas involved in vestibular cognition (Figure 1), then the four probable main pathways transmitting vestibular inputs to those cortices (Figure 2).

## Cognitive functions and vestibular cortices

The vestibular cortical projection areas can be defined as the cortical areas activated during selective stimulation of the vestibular system (e.g., whole body rotation in darkness, excluding visual and proprioceptive stimulation). Studies in rodents, cats, monkeys or humans have identified nine major vestibular cortical areas, most of them playing a specific role in spatial cognition (see Shinder and Taube, 2010 for a review).

### Parieto-Insular Vestibular Cortex (PIVC) and temporo-parietal junction

The Parieto-Insular Vestibular Cortex (PIVC) is usually described as the principal vestibular cortex because 1/3 of its neurons are sensitive to vestibular stimulation (Grüsser et al., 1982; Akbarian et al., 1988; Grüsser et al., 1990a; see Lopez and Blanke, 2011 for review). The PIVC is more precisely located in the lateral sulcus, on its temporal lip in Platyrrhini (e.g., Squirrel Monkey) but on its parietal lip in Catarrhini (e.g., Macaque). In cats the PIVC would be partially represented by the Anterior Supra-Sylvien cortex (see Lopez and Blanke, 2011 for review). In humans the exact location of the PIVC is not clear, but fMRI studies show activation of the temporo-parietal junction (i.e., the superior temporal gyrus, posterior insula, inferior parietal lobule) or more precisely the area OP2 of the parietal opercula (zu Eulenburg et al., 2011; see Lopez and Blanke, 2011 for review). PIVC neurons also receive proprioceptive input, mostly during body movement independent of head movement. This allows the PIVC to integrate body motion in reference to the vestibular inputs (Robinson and Burton, 1980; Akbarian et al., 1988; Grüsser et al., 1990b; Schneider et al., 1993; Björnsdotter et al., 2009; see Shinder and Taube, 2010 for review; Figure 1). This representation of body movement is called idiothetic (i.e., egocentric) because the reference (e.g., vestibular input) is in the body, contrary to allothetic (i.e., allocentric) representations, where the reference is part of the environment (e.g., visual cues). In humans, the temporo-parietal junction could also integrate vestibular input involved in mental rotation tasks in an egocentric reference frame (Falconer and Mast, 2012).

### Anterior parietal cortex

As early as 1966, vestibular input was identified in the anterior parietal somatosensory cortex (Fredrickson et al., 1966). Three different areas have been identified: (1) area 2v is part of area 2, posterior to the somatosensory area of the hand and the mouth (monkey and cat) (Büttner and Buettner, 1978); (2) the area 3aHv is located within the 3a somatosensory field of the hand and arm, at the anterior bank of the central sulcus (squirrel monkey, cat) (Ödkvist et al., 1974); and (3) the area 3aNv is also a part of area 3a, but where the neck is represented and extends anteriorly into the motor cortex (area 4) (Guldin et al., 1992; see Guldin and Grüsser, 1998 for review). About 30 to 50% of the neurons in 3aNv are responsive to vestibular stimuli (see Guldin and Grüsser, 1998 for review). Functional imaging in humans shows activation of the anterior part of the intraparietal sulcus and primary somatosensory cortex, arguing for a human equivalent of area 2v, 3aHv and 3aNv. However, the anterior part of area 7 in humans (7 ant) may also be the homolog of area 2v in the monkey brain (see Brandt and Dieterich, 1999 for review). The anterior parietal vestibular cortex is thought to be a center of integration of vestibular input and somatosensory information from the head, neck and upper limbs. (Ödkvist et al., 1974; Zarzecki et al., 1983; Akbarian et al., 1993; Guldin et al., 1993; Akbarian et al., 1994) This integration possibly plays a role in differentiating self from object motion (see Shinder and Taube, 2010 for review; Figure 1).

### Posterior parietal and medial superior temporal cortices

The posterior parietal cortex contains two major areas involved in vestibular cognition: the ventro-intraparietal cortex and area 7a. The ventro-intraparietal cortex is located in the fundus of the intra-parietal sulcus, neighboring the medial and lateral intraparietal areas (Bremmer, 2005). The area 7a (i.e., area PG) represents the medial part of Brodmann area 7 in the inferior parietal lobule (Andersen et al., 1990). Functional imaging in humans during vestibular stimulation shows activation of the inferior parietal lobule in area 39 and 40, which could therefore correspond to area 7 in monkeys (Bottini et al., 1994; Vitte et al., 1996; Suzuki et al., 2001; see Lopez and Blanke, 2011 for review). Area 7 in monkeys must be differentiated from area 7 in humans, which is also activated by vestibular stimulation but located in the lateral superior parietal lobule (Vitte et al., 1996; Suzuki et al., 2001). The posterior parietal cortex is known as a multimodal center playing a key role in spatial representation and encodes precise self-motion and acceleration states (Andersen, 1997; Whitlock et al., 2012). In the ventro-intraparietal cortex, about half of the neurons receive vestibular input, and almost all of them receive visual input while less than half receive somatosensory input (Bremmer, 2005). From these inputs the ventro-intraparietal cortex creates a representation of space within about 1 meter of the subject and integrates visual object location information relative to the head (e.g., an approaching target to the face) (see Colby and Goldberg, 1999 and Bremmer, 2005 for review). It may also suppress reflex movement during active movement (Klam and Graf, 2006). On the other hand, area 7a in the inferior parietal lobule receives very little vestibular input and contributes to an allocentric representation of visual objects in the environment (Snyder et al., 1998; Chafee et al., 2007; Crowe et al., 2008).

Another allocentric representation of space occurs in the medial superior temporal cortex of monkeys, which detects self-motion from vestibular and visual inputs, and distinguishes them from object motion and updates spatial orientation (Geesaman and Andersen, 1996; Duffy, 1998; Gu et al., 2006; Fetsch et al., 2007; see Shinder and Taube, 2010 for review). In humans the equivalent of the medial superior temporal cortex is probably located in Brodmann area 37 of the middle temporal gyrus (Bense et al., 2001; Stephan et al., 2005; Figure 1).

### Cingulate gyrus and retrosplenial cortex

The “vestibular cingulate region” corresponds to the anterior part of the cingulate gyrus (area 24), showing a strong connection with the PIVC, area 3a and the visual posterior sylvian area in monkeys (Guldin et al., 1992; see Guldin and Grüsser, 1998; Lopez and Blanke, 2011 for reviews). In humans, functional imaging during caloric vestibular stimulation demonstrated activation of the anterior and posterior cingulate gyrus, which are reciprocally connected (Nieuwenhuys et al., 2008). Another study showed activation of the retrosplenial cortex (area 29 and 30) (Vitte et al., 1996), which plays a key role in navigation and path integration (Cooper and Mizumori, 2001; Cooper et al., 2001; Whishaw et al., 2001). The retrosplenial cortex could also transform a representation from allocentric to egocentric (and vice versa) (see Vann et al., 2009 for review; Figure 1).

### Hippocampal and parahippocampal cortices

The hippocampus and parahippocampal area (i.e., entorhinal, perirhinal and postrhinal cortices) integrate cognitive maps (see McNaughton et al., 1996, 2006 for reviews). The construction of these maps is based on place cells, border cells, head direction cells (HD cells) and grid cells which are all predominant in these brain areas. These cell types have been extensively studied, with most work being performed in rodents. Place cells are defined as having an activity highly correlated with the location of the subject in a specific area of the environment (O’Keefe, 1976; Figure 1). They are found in CA1 (pyramidal cells), CA3 of the hippocampus (pyramidal cells), dentate gyrus (granule cells), subiculum (pyramidal cells), parasubiculum, entorhinal and postrhinal cortices (Brown and Taube, 2007). There is some evidence for place cells in the human hippocampus and they are associated with spatial view cells in the parahippocampal region (Ekstrom et al., 2003). However, some of these cells responded to place and view, which makes them substantially different from the usual definition of a place cell. Nonetheless, Ekstrom et al. (2003) estimated that approximately 11% of the recorded cells responded to place but not view and these were most common in the hippocampus. Contrary to place cells, grid cells do not fire in only one location but in multiple specific locations forming an equilateral triangle grid-like pattern (Fyhn et al., 2004; Hafting et al., 2005; Figure 1). Grid cells have been found so far in the lateral and medial entorhinal cortices of rodents and recently humans (Fyhn et al., 2007; Jacobs et al., 2013) and provide a two-dimensional metric for space (Hafting et al., 2005). However, the putative grid cells in humans were demonstrated to exhibit grid-like firing during a virtual navigation task, and therefore this is quite different to the grid cells recorded in rodents during actual spatial navigation (Jacobs et al., 2013). Border cells fire at the boundaries of an environment (Figure 1). They are found in all layers of the medial entorhinal cortex, the parasubiculum and the postsubiculum (PoS; Solstad et al., 2008; Clark and Taube, 2012). The fourth type of cell, the HD cells, are characterized by the highest rate of firing when the head is facing a narrow range of directions. They have been studied mostly in rodents, but also in monkeys and are found in numerous cortical locations including the PoS and CA1 of the hippocampus and also several subcortical nuclei (cf: the pathway from the dorsal tegmental nucleus via the lateral mammillary nucleus, the anterodorsal nucleus of the thalamus to the entorhinal cortex, below).

Vestibular input appears to be fundamentally important for place and HD cells, as inactivation of the vestibular system leads to the disruption of location-specific firing in hippocampal place cells and the direction-specific discharge of thalamic and PoS HD cells (Stackman and Taube, 1997; Stackman et al., 2002; Russell et al., 2003). Moreover, electrical stimulation of different vestibular sensors induces field potentials in the guinea-pig hippocampus (CA1, CA2) (Cuthbert et al., 2000). Also electrical stimulation of the medial vestibular nucleus increases the firing rate of CA1 complex spiking cells (putative place cells) in rats (Horii et al., 2004). In humans, functional imaging during vestibular stimulation demonstrates activation or inactivation of the hippocampal and parahippocampal areas (Bottini et al., 1994; Vitte et al., 1996; Suzuki et al., 2001; Deutschländer et al., 2002; Fasold et al., 2002; Dieterich et al., 2003). Most importantly, patients with chronic bilateral vestibular deficits demonstrate bilateral hippocampal atrophy and spatial memory impairment (Brandt et al., 2005).

All of these results emphasize the fundamental role of vestibular inputs in integrating different maps of the same environment in the hippocampus. The formation of those maps probably depends on the grid cells and some integration of grid cell and HD firing in the entorhinal cortex (McNaughton et al., 2006; Fyhn et al., 2007; Brun et al., 2008; Moser et al., 2008; Figure 2). Activation of those maps depends on current location, environmental context, or current and recent environmental events (McNaughton et al., 1996; Sharp, 1999).

Besides the spatial representation integrated in these maps, place cells contribute to time representation of the past (spatial memory) and the future (navigational planning) (Leutgeb et al., 2005; Pfeiffer and Foster, 2013).

### Other cognitive processes involving vestibular input

Beside spatial cognition, the vestibular system is also suspected to play a role in object recognition and possibly even numerical cognition.

#### Object recognition

Object recognition is impaired in rats, 3 and 6 months after bilateral vestibulectomy (Zheng et al., 2004). These results possibly arise from the loss of vestibular inputs in the entorhinal and perirhinal cortices. Those two cortical areas are indeed involved in object recognition (Mumby, 2001; Winters et al., 2004). Moreover, nitric oxide synthase—an enzyme involved in neuronal plasticity—decreases in the entorhinal and perirhinal cortices 2 weeks after unilateral vestibulectomy (Liu et al., 2004). Nevertheless object recognition was not impaired after sequential chemical vestibulectomy in rats, possibly as a result of partial compensation between the two lesions (Besnard et al., 2012).

The relation between vestibular stimulation and object recognition is probably integrated in place cells, as those cells are responsive to both spatial and non-spatial information, such as geometric and behavioral aspects of the environment (Brown and Taube, 2007).

#### Numerical cognition

The role of vestibular information in numerical tasks was first suspected clinically, after Risey and Briner (1990–1991) reported patients with vertigo who were having difficulties counting backwards by two. This result could be interpreted as an effect of vestibular dysfunction on spatial representation, which seems to play a role in number representation (see for review Smith, 2012).

The relation between numerosity and spatial representation is illustrated by self-motion direction influencing the processing of numbers (Hartmann et al., 2012a). For instance, Hartmann et al. (2012b) showed that passive whole-body motion leftward and downward facilitated small number generation, whereas rightward and upward displacement facilitated the generation of large numbers. In addition, Lugli et al. (2013) showed that passive or active movement modulated the calculation process: addition was facilitated if moving up on an elevator, and subtraction when moving down.

The vestibular system may play a role in these processes, as galvanic vestibular stimulation also influences number generation (Ferrè et al., 2013). Finally, the parietal cortex and more particularly the ventro-intraparietal cortex is involved in number representation and is also one of the vestibular projection areas (Hubbard et al., 2005).

As the role of each of the vestibular cortical projection areas emerges and the integration of vestibular and other sensory information within the hippocampus is better understood, we need to specify the pathways that bring vestibular input to these areas. Most of them involve the thalamus; however, other pathways are possible.

Contrary to most sensory system pathways which reach one specific thalamic nucleus, vestibular input is distributed throughout more than 10 different nuclei (see Lopez and Blanke, 2011 for review).

### Four pathways to the thalamus

Four pathways are known to transmit vestibular inputs to the thalamus: the medial longitudinal fasciculus, the ascending tract of Deiter, the crossing ventral tegmental tract and the ipsilateral vestibulo-thalamic tract (Zwergal et al., 2009). Except for the latter, all of these pathways are involved in vestibulo-ocular function. However, neurons involved in cognition (i.e., vestibular-only neurons) are different from those involved in vestibulo-ocular function (Cullen, 2012).

According to anatomical studies in monkeys, the medial longitudinal fasciculus links the vestibular nuclear complex (VNC) to the contralateral posterior thalamus (Lang et al., 1979; Zwergal et al., 2009). Additionally, studies in rats, cats and monkeys show an ipsilateral connection. Neuronal tracer studies demonstrate that the medial longitudinal fasciculus links: (1) the superior vestibular nucleus to the ipsilateral central lateral nucleus, bilateral ventro-postero-lateral and bilateral ventro-lateral thalamic nuclei; (2) the medial vestibular nucleus to the bilateral ventro-postero-lateral, and the contralateral central lateral thalamic nuclei; and (3) the descending vestibular nuclei to the contralateral medial geniculate nucleus (Lang et al., 1979; Kotchabhakdi et al., 1980; Nagata, 1986; Shiroyama et al., 1999). Moreover, pathologic lesions of the medial longitudinal fasciculus in humans have revealed alterations of the subjective visual vertical (i.e., capacity to orient vertically a bar in the dark) (Zwergal et al., 2008). Therefore, the medial longitudinal fasciculus is probably involved in the vestibular-perception network.

Compared to the medial longitudinal fasciculus, the ascending tract of Deiter links the superior vestibular nucleus and medial vestibular nucleus to the central-lateral, ventral-posterior-lateral and ventral-lateral thalamic nuclei in rats and cats (Kotchabhakdi et al., 1980; Maciewicz et al., 1982; Nagata, 1986; Shiroyama et al., 1999). In monkeys, few projections from the lateral part of the VNC reach the ipsilateral thalamus through the ascending tract of Deiter, the rostral ocular-motor nucleus and the H1 field of Forel (Zwergal et al., 2009). Nevertheless, if the ascending tract of Deiter is involved in oculomotor vergence, no role in vestibular cognition has been demonstrated yet (Zwergal et al., 2009).

Similarly, no cognitive role is proven for the crossing ventral tegmental tract. This vestibulo-oculomotor pathway transmits the anterior canal inputs through the superior vestibular nucleus and Y-group to the contralateral oculomotor nucleus (III), and also the thalamus (anatomical studies in monkey) (Lang et al., 1979; Zwergal et al., 2009).

The fourth vestibular pathway to the thalamus is the ipsilateral vestibulo-thalamic tract, which ascends from the Y-group and probably transmits otolithic signals to the postero-lateral thalamus (Zwergal et al., 2009). Clinical data in humans demonstrates the role of the ipsilateral vestibulo-thalamic tract in the subjective visual vertical; the ipsilateral vestibulo-thalamic tract is understood as a fast pathway transmitting vestibular information to the thalamus and vestibular cortices, which provides it to the cortical multisensory network for the perception of body motion and spatial orientation (Zwergal et al., 2009).

A fifth pathway may involve projections from the bilateral medial vestibular nuclei and the ipsilateral superior and descending vestibular nucleus, to the parafascicular nucleus (PFN) of the thalamus (Lai et al., 2000; see Lopez and Blanke, 2011, for a review). The central lateral and paracentral nuclei also receive vestibular inputs. Although it has not been demonstrated as yet, that the PFN and other intralaminar nuclei (ILN) neurons that receive vestibular input, project to the cortex, this is very likely since the ILN are known to have such projections (see Lopez and Blanke, 2011, for a review).

### Systematization between the vestibular nuclei, thalamus and vestibular cortices

Every vestibular nucleus projects to several or many thalamic nuclei. Most of these projections are contralateral or bilateral (see for review Lopez and Blanke, 2011). Particularly the superior vestibular nucleus and the medial vestibular nucleus send projections to the thalamic ventral posterior complex: i.e., ventral-posterior-lateral nucleus, nucleus ventralis intermedius, ventral posterior medial nucleus or ventral posterior inferior nucleus (in certain species the difference between the ventral posterior medial nucleus and ventral posterior inferior nucleus is not clear) (see Lopez and Blanke, 2011 for review). The superior, medial, lateral and descending vestibular nuclei project to the medial geniculate, the lateral geniculate and the suprageniculate nuclei (Liedgren et al., 1976; Kotchabhakdi et al., 1980; Nagata, 1986; Shiroyama et al., 1999). However, Meng et al. (2007) found a much wider distribution of vestibular responses within the thalamus and reported evidence of cerebellar-thalamic projections which carry vestibular information.

**Figure 3:**
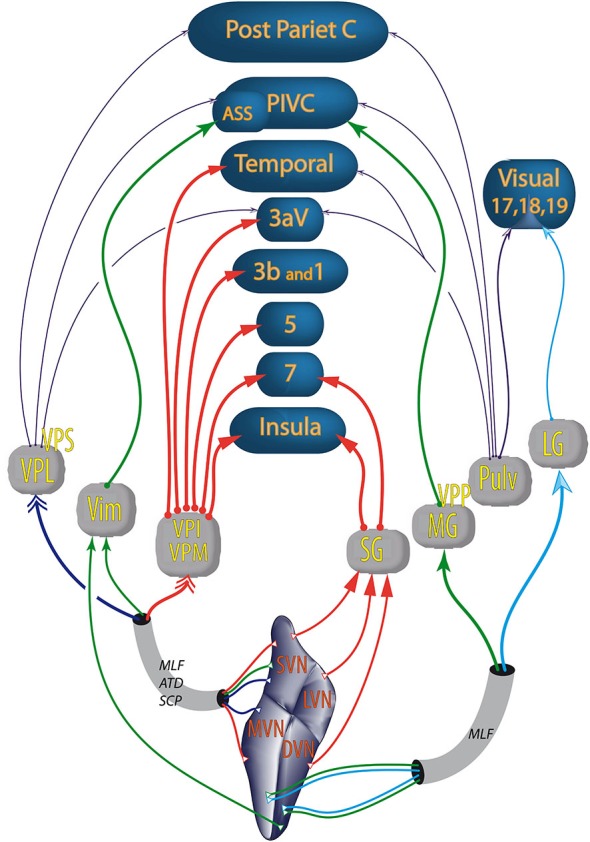
**Vestibular pathways to cortices through the thalamus**. Colors of the arrows differentiate the thalamic nuclei. Double arrows (→>) correspond to bilateral projections. The other types and thickness of the arrows are varied to improve the readability of the figure; ATD, ascendant tractus of Deiters; ASS, anterior suprasylvian cortex (homolog in cats of the primate PIVC); DVN, descending vestibular nuclei; LG, lateral geniculate; LVN, lateral vestibular nuclei; MLF, medial longitudinal fasciculus; MG, medial geniculate nucleus; MVN, medial vestibular nuclei; PIVC, parieto insular vestibular cortex (in primates); Post Pariet C, posterior parietal cortex; Pulv, pulvinar; SG, suprageniculate nucleus; SCP, superior cerebellar pedunculus; SVN, superior vestibular nuclei; Temporal, temporal cortex; Vim, nucleus ventralis intermedius; VPI, ventral posterior inferior nucleus; VPL, ventral posterior lateral nucleus; VPM, ventral posterior median nucleus; VPP, nucleus ventralis posterior pars posterior; VPS, ventral posterior superior nucleus; numbers (e.g., 5 or 3b) refer to Brodmann areas. From studies in rodents, cats and primates (Nagata, 1986; Akbarian et al., 1992; Reep et al., 1994; Marlinski and McCrea, 2008; Lopez and Blanke, 2011).

Among the thalamic nuclei, some neurons are responsive only to vestibular stimulation (i.e., first order relay), for example, the ventral posterior complex (Marlinski and McCrea, 2008). Other vestibular thalamic nuclei are higher order relays which are also sensitive to somatosensory (e.g., ventral-posterior-lateral, ventral-posterior-medial, ventral-posterior-inferior nuclei) or visual inputs (e.g., lateral geniculate nucleus) (Reichova and Sherman, 2004; Sherman, 2007; Sherman and Guillery, 2011). Vestibular thalamic nuclei project to primary somatosensory (area 3aV), visual (area 17) cortices and the polymodal parietal, temporal and insular cortices involved in spatial cognition (see Liedgren et al., 1976; Kotchabhakdi et al., 1980; Nagata, 1986; Akbarian et al., 1992; Reep et al., 1994; Shiroyama et al., 1999; Marlinski and McCrea, 2008; Figure 3). Some authors distinguish inside the ventroposterior thalamus a nucleus ventralis posterior superior (VPS) and a nucleus ventralis posterior pars posterior (VPP; Akbarian et al., 1992; Marlinski and McCrea, 2008). According to these authors, vestibular sensitive neurons from the VPS project to area 3aV, those from the VPP project to the PIVC and those from the oral and medial pulvinar to area T3.

The anatomical pathways from the thalamus to the cortices probably go through the three thalamic peduncles: the superior and posterior peduncles reaching the central parietal and occipital cortices; and the inferior thalamic peduncle, reaching the orbito-frontal, insular and temporal cortices (Nieuwenhuys et al., 2008).

### Pathway to the hippocampus

**Figure 4:**
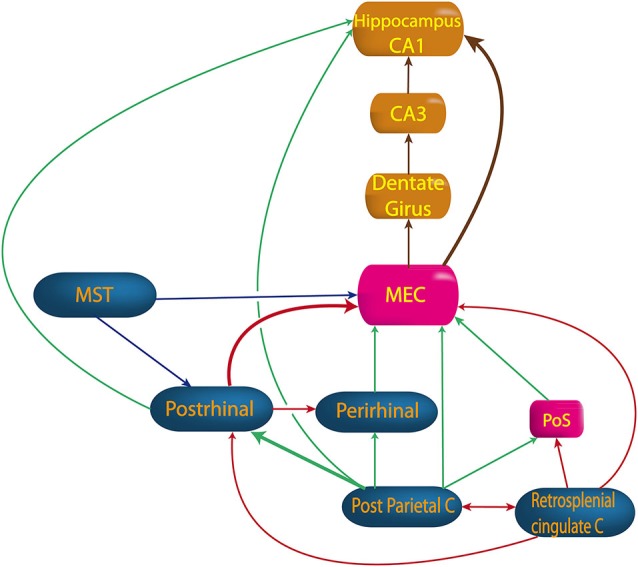
**Pathway from vestibular cortices to hippocampus**. MEC, medial entorhinal cortex; MST, medial superior temporal cortex; Perirhinal, perirhinal cortex; PoS, posterior subiculum; Postrhinal, postrhinal cortex; Retrosplenial cingulate C, retrosplenial and cingulated cortices. (built from Burwell and Amaral, 1998; Rockland and Van Hoesen, 1999; Moser et al., 2008; Shinder and Taube, 2011).

No direct projection from the VNC or thalamus to the hippocampus has ever been proven. Hence, the vestibular cortices are a good candidate to send vestibular inputs to the parahippocampal area (Figure 4). Most evidence concerns the posterior parietal cortex, including area 7a, which sends projections to the hippocampal CA1 area (anatomical studies in monkey) (Rockland and Van Hoesen, 1999). Additionally, another pathway, the “head direction pathway”, reaches the hippocampus through the medial entorhinal cortex (MEC; Aggleton et al., 2000). The MEC contains place cells, grid cells and HD cells (see Taube, 2007; Moser et al., 2008 for reviews) and 16% of its inputs come from vestibular cortices involved in visuospatial function (i.e., posterior parietal cortex, cingulate and retrosplenial cortices). The parietal cortex itself represents 9% of the MEC’s inputs. The posterior parietal cortex projects to the MEC, either directly or mostly through the postrhinal cortex (parahippocampal cortex in monkey) (Burwell and Amaral, 1998). Other indirect projections from the posterior parietal cortex to the MEC go through the perirhinal cortex (mostly area 36 rather than 35) or the PoS (Moser et al., 2008; Shinder and Taube, 2010 for reviews). However, these last two sources of inputs are minor compared with the postrhinal cortex, in terms of their anatomical connections and their visuospatial functions (Burwell and Amaral, 1998; Aggleton et al., 2000). From the MEC (layer II), extensive projections reach the dentate gyrus then CA3 and CA1 (Burwell and Amaral, 1998; Brun et al., 2008). However, some direct connections also exist from the MEC (layer III) or from the postrhinal cortex to the hippocampus (Burwell and Amaral, 1998; see Moser et al., 2008 for review). These direct projections from layer III are more important for the accuracy of place fields in CA1 than the projections from layer II (Brun et al., 2008).

Other projections to the MEC come from the cingulate and the retrosplenial cortices, either directly or through the postrhinal cortex or the dorsal presubiculum (Burwell and Amaral, 1998; see Shinder and Taube, 2010 for review). Finally, projections also exist from the medial superior temporal area either directly or through the postrhinal cortex (see Shinder and Taube, 2010 for review).

The MEC seems to play a key role in spatial cognition as hippocampal place cells are related to entorhinal border cells, HD cells, and grid cells which determine place fields in the hippocampus (Brun et al., 2008; Zhang et al., 2013). However, abolition of the head direction signals in the antero-dorsal nucleus of the thalamus (ADN) and the dorsal presubiculum significantly degraded place cell responses in CA1 (Calton et al., 2003), suggesting that the vestibulo-thalamo-cortical pathway may not be independent of the head direction pathway.

## The pathway from the dorsal tegmental nucleus to the entorhinal cortex

Beside a representation of position (place cells), a representation of direction (HD cells) is essential to navigate without landmarks (e.g., an unknown environment) (see Etienne and Jeffery, 2004 for review). Vestibular inputs are fundamental to head direction representation, especially when visual cues are absent (e.g., in darkness or in a desert) (Blair and Sharp, 1996). Consequently, the HD cell signal is abolished in the ADN after bilateral vestibulectomy by arsanilate, and also in the PoS after intratympanic injection of tetrodotoxin (Stackman and Taube, 1997; Stackman et al., 2002). Vestibular input is fundamental as the HD signal does not recover even 3 months after the lesion, showing that other sensory inputs are not sufficient to compensate for the loss of vestibular signals (Clark and Taube, 2012). Both semicircular canals and otoliths play a role as the HD signal is disrupted after canal occlusion (Muir et al., 2009) and is unstable in otolithic deficient mice (Beraneck and Lambert, 2009; Yoder and Taube, 2009).

### Location of the HD signal

Although HD cells were first described in the PoS (Ranck, 1984; Taube et al., 1990), they have been found since in all the areas of the limbic system: the lateral mammillary nuclei (Stackman and Taube, 1998), anterior dorsal thalamic nucleus (Taube and Burton, 1995), retrosplenial cortex (Chen et al., 1994), entorhinal cortex (Sargolini et al., 2006) and CA1 (Leutgeb et al., 2000). However, they are also found in non-limbic areas, such as the dorsal tegmental nucleus, the lateral dorsal thalamus (Mizumori and Williams, 1993), the dorsal striatum (Wiener, 1993; Mizumori et al., 2000), the medial prefrontal cortex (i.e., FR2 or AGm cortex) (Taube, 2007), and the medial prestriate cortex (Chen et al., 1994).

The concentration of HD cells in each of these areas varies from 60% of the cells in the ADN (Taube and Burton, 1995), 30% in the lateral dorsal thalamus (Mizumori and Williams, 1993), 25% in the PoS and lateral mammillary nucleus, 12% in the dorsal tegmental nucleus (Sharp et al., 2001), 10% in the retrosplenial cortex to 6% in the striatum (Mizumori et al., 2000).

### Angular head velocity cells

In addition to the classical HD cells, some cells fire in relation to the speed and direction when an animal turns its head. These “Angular Head Velocity” cells (AHV) are found mostly in the dorsal tegmental nucleus (75% of the cells) (Bassett and Taube, 2001), in the lateral mammillary nucleus (50% of the cells) (Stackman and Taube, 1998) and also in the lateral habenula (Taube, 2007).

### Organization of the HD pathway

**Figure 5:**
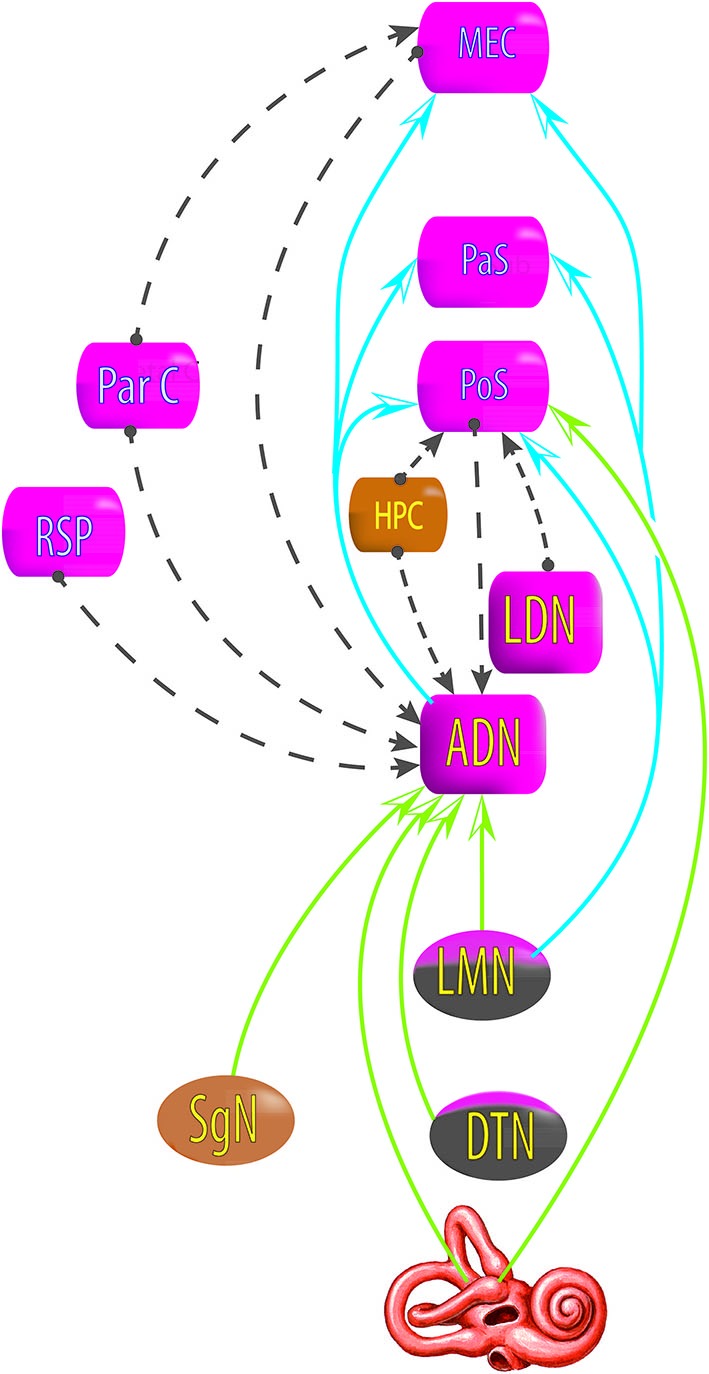
**Relationship between areas exhibiting head direction signals, deduced from lesion studies**. Blue and green arrows: lesions at the starting area disrupt the head direction signal in the end area. Gray dashed line arrows: lesions at the starting area do not disrupt head direction signal in the end area. DTN, dorsal tegmental nucleus; LMN, lateral mammillary nucleus; AND, antero dorsal nucleus of the thalamus; LDN, latero dorsal thalamus; HPC, hippocampus; PoS, posterior subiculum; RSP, retrosplenial cortex; ParC, partietal cortex; PaS, parasubiculum; MEC, medial entorhinal cortex (see review in Clark and Taube, 2012).

Several lesion studies have deduced the pathways connecting areas with HD cells (Figure 5). Bilateral lesions of vestibular labyrinth, the dorsal tegmental nucleus or the lateral mammillary nucleus disrupt the HD signal in the ADN (Stackman and Taube, 1997; Blair et al., 1998; Bassett et al., 2007). Additionally, lesions of the ADN or the lateral mammillary nucleus disrupt the HD signal in the PoS, the MEC and the parasubiculum (Goodridge and Taube, 1997; Blair et al., 1998; Bassett et al., 2007; Sharp and Koester, 2008; Clark and Taube, 2012).

However, the HD signal in the ADN is not disrupted by lesions of the PoS, nor lesions of the hippocampus, the retrosplenial cortex, the parietal cortex, or the MEC (Golob and Taube, 1997; Calton et al., 2008; Clark et al., 2010; Clark and Taube, 2012); and lesions of the parietal cortex do not disrupt the HD signal in the MEC (Clark and Taube, 2012); lesions in the hippocampus do not disrupt the HD signal in the PoS (Golob and Taube, 1997, 1999) and lesions in the PoS do not disrupt the HD signal in the lateral mammillary nucleus (Clark and Taube, 2012).

**Figure 6:**
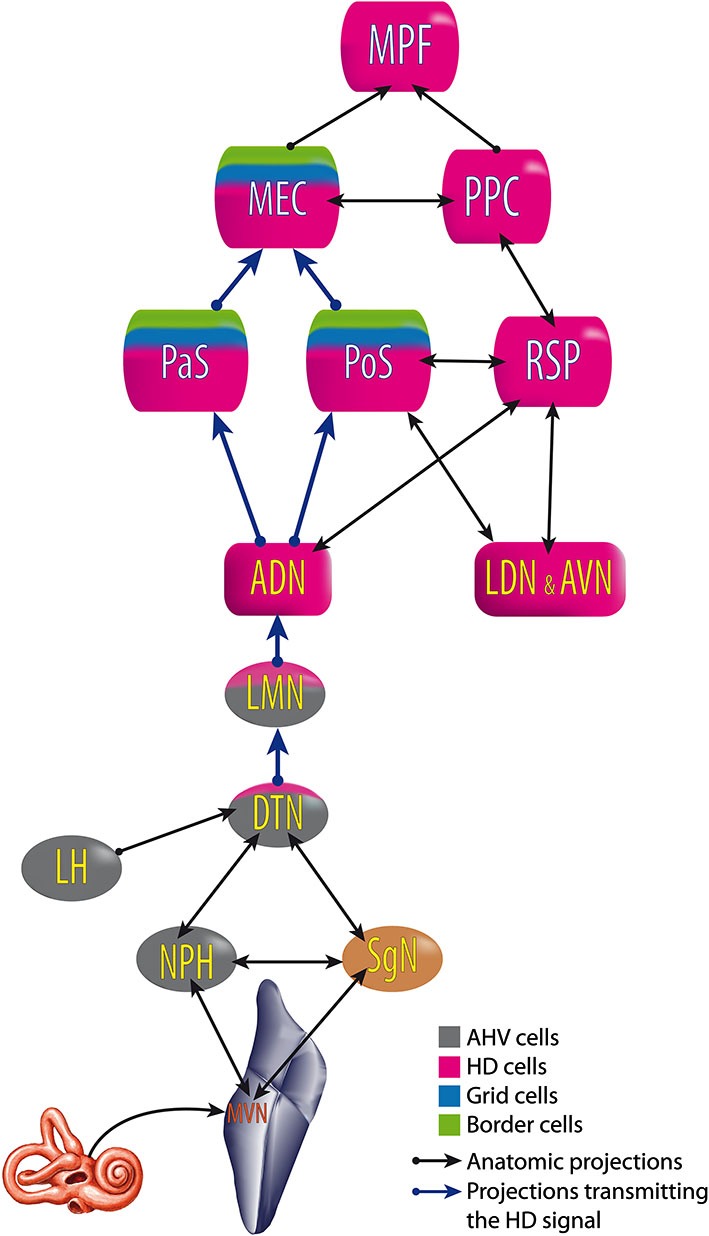
**Head Direction pathway**. ADN, anterodorsal thalamus; AVN, anteroventral thalamus; DTN, dorsal tegmental nucleus; LDN, laterodorsal thalamus; LMN, lateral mammillary nuclei; MEC, medial entorhinal cortex; MPF, medial prefrontal cortex; MVN, medial vestibular nuclei; NPH, nucleus prepositus hypoglossi; PaS, parasubiculum; PoS, postsubiculum; PPC, posterior parietal cortex; RSP, retrosplenial cortex; SgN, supragenual nucleus. Built from Hoover and Vertes (2007); Taube (2007) and Clark and Taube (2012).

All of these results suggest that the HD signal from the vestibular nuclei is transmitted to the dorsal tegmental nucleus, then the lateral mammillary nucleus, the ADN and finally the PoS, parasubiculum and MEC. Nevertheless, no direct anatomical pathways are described to explain the transmission of vestibular input to the dorsal tegmental nucleus (Taube, 2007). However, indirect pathways are known through the nucleus prepositus hypoglossi or the supragenual nucleus (Liu et al., 1984; McCrea and Baker, 1985; Graf et al., 2002; Biazoli et al., 2006). No HD nor AHV cells are described in these nuclei, but the prepositus hypoglossi prolongs the phase of the vestibular signals (beyond 90° relative to velocity) compared to the signal in the vestibular nerve i.e., “mathematical integration” towards 180° out of phase with velocity (Taube, 2007). This could play a role in the HD signal generation. Besides, the supragenual nucleus seems essential for the HD signal as bilateral lesions of it significantly reduce the number of HD cells in the ADN (Clark et al., 2012; Figure 6).

## The pathway from the nucleus reticularis pontis oralis, to the medial septum to the hippocampus

Hippocampal theta rhythm (theta) is an oscillating electrical signal within the 4–10 Hz frequency range found in rodents (rabbit, mice, rat), but also dogs and cats. Theta at this frequency is almost absent in monkeys, and rare or absent in humans (Niedermeyer, 2008) but the equivalent of theta in humans could be in fact much slower (1–4 Hz) (Jacobs, 2014). Theta rhythm can be found in the dentate gyrus and CA1 of the hippocampus, but also in the subiculum, the entorhinal cortex, the cingulate cortex, the mammillary bodies, the posterior hypothalamus, the amygdala and the prefrontal cortex (O’Keefe, 1993; Niedermeyer, 2008; Hartley et al., 2014).

Cognitive functions like spatial orientation or spatial memory require theta rhythm in the hippocampus (Leutgeb et al., 2005), probably because theta establishes a subthreshold membrane potential and modulates the spiking activity of hippocampal, entorhinal, and septal neurons endowed with voltage-dependent channels (Leung and Yim, 1986; Vertes and Kocsis, 1997; Buzsáki, 2002; Lubenov and Siapas, 2009).

### Influence of vestibular input on theta

Several authors have argued that vestibular input can influence theta rhythm. For instance, passive rotation of awake restrained rats increases theta power in both light and complete darkness and this increase appeared with vestibular nystagmus (Gavrilov et al., 1995, 1996). Theta power also increases during passive translation in rats but less so during rotation (Gavrilov et al., 1996). More precisely, the theta rhythm induced by rotation is sensitive to atropine (Type 2 Theta) (Shin, 2010) and most probably linked to the cholinergic neurons of the medial septum (Tai et al., 2012). In cats and dogs, passive translation does not increase the power but increases the peak frequency of theta (Arnolds et al., 1984). Moreover, vestibular lesions decrease the power and the frequency of theta (Russell et al., 2006; Neo et al., 2012; Tai et al., 2012). Nevertheless, restoration of theta in vestibular-deficient rats, by medial septum stimulation, is not sufficient to compensate for cognitive impairment induced by vestibular lesions (Neo et al., 2012).

### Theta pathway

Structures involved in theta rhythm include the reticularis pontis oralis, the pedonculopontine tegmental nucleus (PPTg), the supramammillary nucleus, the posterior hypothalamus, the septal complex, the entorhinal cortex and the hippocampus (Vertes and Kocsis, 1997; Bland and Oddie, 1998; Pignatelli et al., 2012).

During theta, ascending signals from the reticularis pontis oralis activate neurons of the supramammillary nucleus; the supramammillary nucleus, in turn, converts this steady barrage of action potentials into a rhythmical pattern of discharge which is relayed to GABAergic/cholinergic rhythmically-bursting cells of the medial septum. The septal rhythmically bursting cells modulate subsets of hippocampal interneurons and principal cells in the generation of theta rhythm (see for review Vertes and Kocsis, 1997). A reduction of theta oscillation in the medial septum has been reported to disrupt the spatial selectivity of grid cells but not place cells or HD cells (Brandon et al., 2011; Koenig et al., 2011).

In this system the PPTg appears to modulate the reticularis pontis oralis activity through direct cholinergic projections (Shiromani et al., 1988; Semba and Fibiger, 1992; Vertes and Kocsis, 1997). Vestibular inputs are known to project to both the PPTg and reticularis pontis oralis (Vertes and Kocsis, 1997; Bland and Oddie, 1998; Seemungal et al., 2010; Aravamuthan and Angelaki, 2012). The influence of the vestibular system seems important as 72.5% of the PPTg’s neurons in monkeys respond to vestibular stimulation (rotation or translation) (Aravamuthan and Angelaki, 2012).

Besides the PPTg, the vestibular system could influence theta rhythm through the ventral tegmental nucleus of Gudden. This pontic nucleus generates a bursting rhythmic activity that precedes theta activity in the hippocampus (Bassant and Poindessous-Jazat, 2001). Moreover, the ventral tegmental nucleus projects extensively to the median mammillary body involved in the limbic system. Consequently, lesions of the ventral tegmental nucleus impair spatial learning and memory in rats and humans (Vann, 2009). The role of vestibular input in this system has never been studied, but the medial, lateral and superior vestibular nuclei project to the ventral tegmental nucleus, as shown by neuronal tracer studies in mice, rats and cats (Irle et al., 1984). Theta rhythm is also found in the cerebellum (lobule HVI and interposed nucleus) where it is synchronized with hippocampal theta (Hoffmann and Berry, 2009; Wikgren et al., 2010). This phenomenon is thought to enhance associative learning abilities (Hoffmann and Berry, 2009).

## A possible pathway via the cerebellum to the parietal cortex?

### Role of the cerebellum in spatial orientation

The role of the cerebellum in spatial orientation is evident in both humans and animals (see for review Rochefort et al., 2013). The cerebellum is activated on fMRI during virtual navigation (Moffat et al., 2006); and patients with cerebellar lesions suffer from visuospatial, linguistic and affective impairment known as the Cerebellar Cognitive Affective Syndrome (Schmahmann and Sherman, 1998; Middleton and Strick, 2000; Schmahmann, 2001; Partridge et al., 2010). According to clinical cases, the visuospatial function could be located in the nucleus dentate and cerebellar hemispheres (Schmahmann, 1996, 2004).

Other proof of cerebellar involvement in cognition comes from several types of cerebellar mutant mice showing impaired spatial learning, especially if Purkinje cells are deficient (Mullen et al., 1976; Goodlett et al., 1992; Hilber et al., 1998; Rondi-Reig and Burguière, 2005). Additionally, rats with lesions of the pontine nuclei-granule cell-parallel pathway—which transmits vestibular input to the cerebellar cortex—fail to learn the spatial task of the non-visual water-maze, contrary to rats with lesions of the climbing fibers (Rondi-Reig et al., 2002; Barmack, 2003).

Finally, vestibular input reaching the nodulus (cerebellum lobule 10) and uvula (lobule 9) changes from an egocentric representation to an allocentric representation in the Purkinje cells recorded in monkeys (Yakusheva et al., 2007; Angelaki et al., 2010). The rostral fastigial nucleus, on the other hand, integrates vestibular and proprioceptive input to integrate body motion in an egocentric head-centered-reference frame and vestibular signals from a head- to a body-centered-reference frame (Brooks and Cullen, 2009).

### Connections from the vestibular system to the cerebellum

The cerebellum receives direct projections from the vestibular nerve, bypassing the VNC. Most of these projections (>70%) terminate in the nodulus and uvula as mossy fibers (Korte and Mugnaini, 1979; Angelaki et al., 2010). Other vestibular projections come from the VNC (i.e., the superior, medial and descending nuclei, group Y). The VNC projects bilaterally to the flocculus, the fastigial nucleus, the anterior and posterior interposed nuclei and the posterior vermis (mostly the uvula (lobule 9), but also the declive, folium, tuber, and pyramide (lobules 6, 7 and 8)) (Carpenter et al., 1972; Kotchabhakdi and Walberg, 1978; Blanks et al., 1983; Brodal and Brodal, 1985; Walberg and Dietrichs, 1988; Thunnissen et al., 1989; Epema et al., 1990).

### Connections from the cerebellum to the hippocampus

Direct connections from the cerebellum to the hippocampus are suspected, as stimulation of the rostral vermis, fastigial nucleus, and intervening midline folia of the cerebellum, inhibit hippocampal activity with a short latency (Maiti and Snider, 1975; Heath et al., 1978; Newman and Reza, 1979).

Additionally, lesions of the fastigial nucleus induce bilateral degeneration in the hippocampal formation, including CA2, CA3, dentate gyrus and subiculum. Any pathway from the cerebellum to the hippocampus seems likely to go through the fimbria or the dorsal fornix, presubiculum and subiculum (Heath and Harper, 1974).

No direct connections are known between the nodulus or uvula and the hippocampus, but indirect connections are hypothesized (e.g., through the fastigial nucleus) (Yakusheva et al., 2013).

### Connections from the cerebellum through the thalamus

Vestibular input from the fastigial, dentate and interposed nuclei project to the thalamus, mostly in the ventro-lateral nucleus (VLN), but also the ventro-postero-lateral and the medio-dorsal nucleus (paralamellar portion) (Haroian et al., 1981; Angaut et al., 1985; Aumann et al., 1994). The VLN receives vestibular input from the cerebellum in monkeys and both the cerebellum and VNC in rats and cats (Kotchabhakdi et al., 1980; Maciewicz et al., 1982; Nagata, 1986; Shiroyama et al., 1999; Meng et al., 2007).

Anatomical studies show connections from the VLN to three parietal vestibular cortices involved in cognition: the area 3aV, the area 2v (Morecraft et al., 1993) and the posterior parietal cortex (Amino et al., 2001). Moreover, electrical stimulation of the dentate, interposed or fastigial nuclei induces field potentials in the posterior parietal cortex of monkeys, demonstrating a functional link between the cerebellum and the vestibular cortex (Amino et al., 2001).

## New hypothetical pathway to the basal ganglia

### Role of the basal ganglia in spatial cognition

Besides the hippocampus, growing evidence suggests the basal ganglia as a key center for spatial cognition (for review see Mizumori et al., 2009; Retailleau et al., 2012).

The ventral striatum (nucleus accumbens) is involved in both short-term spatial learning and long-term spatial memory as demonstrated by inhibiting striatal glutamate receptors (NMDA and AMPA), or interfering with transcription factors (i.e., CREB) or protein synthesis (Atallah et al., 2006; Ferretti et al., 2007, 2010). Moreover, behavioral studies demonstrate an allocentric spatial representation in the ventral striatum and the postero-dorsomedial striatum, compared with egocentric representation in the dorso-lateral striatum which contains HD cells (Wiener, 1993; Ferretti et al., 2007; Retailleau et al., 2012).

### Role of vestibular input in the cognitive functions of the basal ganglia

Since the early 1970’s, electrophysiological studies have demonstrated responses in the caudate nucleus in response to electrical stimulation of the vestibular nerve (Potegal et al., 1971; Liedgren et al., 1976; cats and squirrel monkeys) or vestibular nucleus (Spiegel et al., 1965; cats). Field potential responses were also obtained in the putamen (Spiegel et al., 1965; Liedgren et al., 1976). However, in alert rhesus monkeys, Matsunami and Cohen (1975) could obtain responses only in the caudate nucleus and globus pallidus at stimulus amplitudes that evoked body movement and therefore it was unclear whether the response was to vestibular stimulation itself. Other evidence for a vestibular-striatal connection comes from vestibular-deficient mutant mice which exhibit an increase in pCREB—a protein involved in spatial memory consolidation—specifically in the striatum (Ferretti et al., 2010; Antoine et al., 2013). Moreover, an anatomical pathway has been demonstrated between the medial vestibular nucleus and the dorsolateral striatum in rats, going through the parafascicular thalamic nucleus (Nagata, 1986; Shiroyama et al., 1999; Lai et al., 2000).

### Striatum-hippocampus connections

The striatum is interconnected with the hippocampus (Scatton et al., 1980; Gasbarri et al., 1994; Floresco et al., 2001; van der Meer et al., 2010). The spatial representation in these two areas is used to perform different types of navigation based on either procedural memory for the striatum, or declarative memory for the hippocampus. Vestibular input influences the strategy of navigation, as rats with bilateral vestibulectomy use a procedural response compared with controls which use procedural and declarative memory-based navigation equally (Machado et al., 2014).

## Conclusion

The knowledge of the anatomical bases of vestibular contributions to cognition has significantly increased in the past decade, suggesting four pathways and perhaps a fifth one through the striatum. Particularly the role of vestibular input in the pathway from the dorsal tegmental nucleus via the lateral mammillary nucleus, the anterodorsal nucleus of the thalamus to the entorhinal cortex, is better understood as well as the organization of the nuclei along this pathway. Nevertheless, some major questions remain such as how and where is the HD signal processed, why do HD cells exist in so many brain areas and what role do they play in each area? The lack of specific cells, such as HD cells, along the other pathways make them more difficult to study and the role of each pathway more difficult to identify. Moreover, several pathways probably interact with each other. For example, the suppression of the HD signal does not suppress the place cell signal in the hippocampus (independent pathways) but significantly degrades it (interaction of the pathways) (Calton et al., 2003). All of the pathways may also interact in the different vestibular cortices or in the hippocampal formation and some of them in the thalamus as well.

Another level of complexity occurs as vestibular inputs are integrated with other sensory inputs in the VNC, which makes them more difficult to isolate. Additionally, the complexity increases as the vestibular signals are disseminated throughout the four pathways described, but also within some pathways (before the thalamus for example in the vestibulo-thalamo-cortical pathway). This wide dissemination of vestibular signals could result from an evolutionary process to elaborate a neural network with sparse coding (Olshausen and Field, 2004; Niven and Laughlin, 2008). Indeed sparse coding is thought to provide a sensory system network at a low energy cost, with a large storage capacity, rapid learning ability, and tolerance to degradation of the network or noise in the input (Olshausen and Field, 2004; Waydo et al., 2006; Quiroga et al., 2008).

To address this complexity, research in vestibular cognition could benefit from new techniques in anatomy (Chung et al., 2013), in electrophysiology (e.g., optogenetic methods), new behavioral tests able to discriminate different cognitive strategies and improvements in functional imaging. Those efforts will increase the probability of better understanding how the vestibular system evolved, what role it plays in cognitive function and how vestibular pathology can impair cognitive functions. Do these cognitive impairments compensate, and how can we treat them?

## Conflict of interest statement

The authors declare that the research was conducted in the absence of any commercial or financial relationships that could be construed as a potential conflict of interest.

## Abbreviations

ADN: Anterior Dorsal Nucleus of the thalamus
AHV: Angular Head Velocity cells
HD cell: Head Direction cell
MEC: Medial Entorhinal Cortex
PIVC: Parieto-Insular Vestibular Cortex
PoS: Postsubiculum = dorsal portion of the presubiculum
PPTg: Pedunculopontine Tegmental Nucleus
Theta: hippocampal theta rhythm
VPS: nucleus ventralis posterior superior (thalamus)
VPP: nucleus ventralis posterior pars posterior (thalamus)
VNC: Vestibular Nuclear Complex.
